# Effects of Printing Orientation on the Tensile, Thermophysical, Smoke Density, and Toxicity Properties of Ultem^®^ 9085

**DOI:** 10.3390/polym17020145

**Published:** 2025-01-09

**Authors:** Elīna Vīndedze, Tatjana Glaskova-Kuzmina, Didzis Dejus, Jānis Jātnieks, Scott Sevcik, Irina Bute, Jevgenijs Sevcenko, Stanislav Stankevich, Sergejs Gaidukovs

**Affiliations:** 1 AM Craft, Braslas 22D, LV-1035 Riga, Latvia; tatjana.glaskova-kuzmina@lu.lv (T.G.-K.); didzis@am-craft.com (D.D.); janis@am-craft.com (J.J.); scott@am-craft.com (S.S.); 2 Institute for Mechanics of Materials, University of Latvia, Jelgavas 3, LV-1004 Riga, Latvia; irina1bute@gmail.com (I.B.); jevgenijs.sevcenko@lu.lv (J.S.); stanislavs.stankevics@lu.lv (S.S.); 3 Institute of Chemistry and Chemical Technology, Faculty of Natural Sciences and Technology, Riga Technical University, P. Valdena 3, LV-1048 Riga, Latvia; sergejs.gaidukovs@rtu.lv

**Keywords:** tensile properties, thermophysical properties, smoke density, toxicity, Ultem 9085, 3D printing, printing orientation, fused filament fabrication (FFF)

## Abstract

Despite the impressive properties of additively manufactured products, their inherent anisotropy is a crucial challenge for polymeric parts made via fused filament fabrication (FFF). This study compared the tensile, thermophysical, smoke density, and toxicity characteristics of Ultem 9085 (a blend of polyetherimide and polycarbonate) for samples printed in various orientations (X, Y, and Z). The results revealed that mechanical properties, such as elastic modulus and tensile strength, significantly differed from the Z printing orientation, particularly in the X and Y printing layer orientations. Thermomechanical analysis revealed that Ultem 9085 had high anisotropic effects in the coefficient of thermal expansion, indicating superior thermal properties along the printing orientation. The smoke density and toxicity test results proved that Ultem 9085 complies with aviation safety standards. Smoke density tests showed that all samples, regardless of print orientation or thickness, stayed well below the regulatory limit, making them suitable for aircraft interiors.

## 1. Introduction

Additive manufacturing has gained widespread adoption across various industries due to numerous advantages, including increased design flexibility, reduced material waste, rapid prototyping, tailored customization, streamlined supply chains, reduced tooling and assembly requirements, and environmental benefits [1]. These advantages have increased the use of additive manufacturing in aerospace, automotive, healthcare, defence, and military fields, offering improved efficiency, cost savings, and innovative product development opportunities [2]. For instance, various 3D-printed polymer materials are utilized in aerospace to meet rigorous industry standards, including high strength-to-weight ratios, resistance to high temperatures, and fire resistance.

However, their notable anisotropy is critical for polymeric parts produced via fused filament fabrication (FFF). This anisotropy primarily arises from the properties of extruded filaments, the layer-by-layer construction process, and the limited bonding between layers [3]. As a result, 3D-printed polymer parts exhibit directional differences in several properties—including flame retardancy [4], mechanical strength [3,5], and thermophysical stability [6]—especially in the direction perpendicular to the layering (XZ plane). Moreover, a significant drawback of FFF is the formation of voids usually observed for 3D printed parts, worsening their mechanical and functional properties [5,7,8].

Moreover, the mechanical, thermophysical, and other physical properties of polymer blends in 3D printing are significantly influenced by the type, ratio, and interaction of their constituents [9,10]. For instance, a well-balanced blend can improve toughness without excessively compromising stiffness [11], but blends of polymers with significantly different glass transition temperatures or melting points can result in tailored thermal properties, ideal for applications requiring thermal resistance or flexibility [12], while a low-viscosity polymer in the blend can improve melt flow during extrusion, enhancing printability [8].

Besides mechanical and thermophysical properties, smoke and toxicity characteristics are crucial in aviation applications because materials used in aircraft interiors, structural components, and other critical areas must meet stringent safety standards to protect passengers and crew [13,14,15]. Aviation authorities, including the Federal Aviation Administration and the European Union Aviation Safety Agency, have strict regulations requiring materials to meet low smoke and toxicity thresholds. Materials in the cabin, cockpit, and other areas must pass fire safety tests, including limits on smoke density and the release of harmful gasses, to ensure regulatory compliance and passenger safety. By understanding materials’ smoke and toxicity characteristics, engineers and designers can make informed decisions when selecting materials that meet performance and safety requirements. This is particularly important as the aviation industry moves toward lighter, advanced materials, such as high-performance polymers for 3D-printed components [16].

Ultem 9085^®^ (a blend of polyetherimide, PEI, and polycarbonate, PC) is a high-performance thermoplastic widely used in aviation due to its exceptional strength-to-weight ratio, flame resistance, and low smoke toxicity [17]. This material is ideal for 3D printing applications, especially in aircraft interiors and components requiring durability and compliance with stringent safety standards. Its inherent flame-retardant properties meet the FAR 25.853 standard, making it suitable for parts that must withstand high temperatures and resist ignition [18]. Additionally, its low density contributes to fuel efficiency, a critical consideration in aerospace design. The material’s compatibility with FFF technology also facilitates fast prototyping and the development of complex geometries, enabling manufacturers to streamline the development of customized, lightweight parts that meet strict regulatory standards in aviation [2,3,4,5,6].

Since Ultem 9085 is a relatively new material, there are some gaps in the research results, mostly in finding the correlation for the anisotropy in different physical properties. As mechanical properties are greatly affected by printing parameters, their optimization results are broadly reported [19,20], with a particular focus on the effect of printing orientation on the mechanical properties of Ultem 9085 printed parts [5,8,21]. The thermophysical properties of Ultem 9085 have been investigated less, particularly regarding the effects of different printing orientations [5,8,22]. According to data in the literature, the least attention is paid to the effect of printing parameters on the flame-retardant, smoke, and toxicity properties of Ultem 9085 [6,23].

This study investigates the anisotropic effects introduced by the FFF process on the tensile, thermophysical, smoke density, and toxicity properties of Ultem 9085 (PEI and PC). The samples were printed and studied in three orthogonal printing orientations: X, Y, and Z. The primary component of Ultem 9085 is PEI, and PC is added to improve the material flow required for additive manufacturing [5,8]. Since the relative content of both constituents in Ultem 9085 is unclear and is usually reported as consisting of only PEI [18,20,24], it was not the purpose of this study to examine the effect of each constituent on the studied properties.

## 2. Materials and Methods

### 2.1. Materials

**Figure 1:**
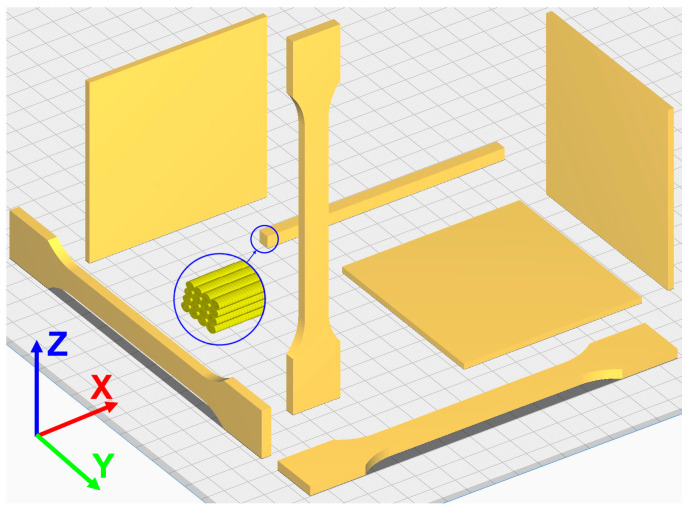
Scheme of the samples used for tensile, thermophysical, smoke density, and toxicity testing.

**Figure 2:**
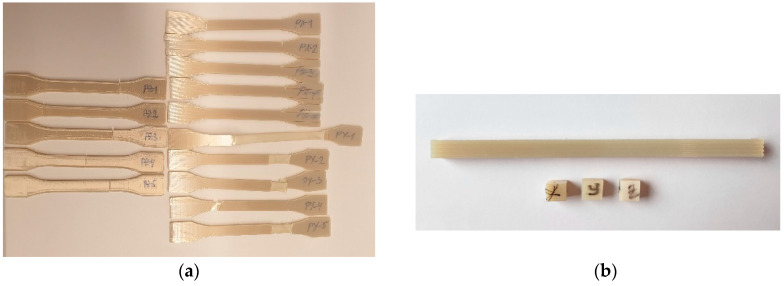
Photos of the test samples used for the (**a**) tensile and (**b**) thermomechanical tests.

The investigated material, Ultem 9085 CG (Certified Grade), was provided by Stratasys (Eden Prairie, MN, USA) and used at AM Craft to produce test samples for tensile, thermophysical, smoke density, and toxicity tests from the same batch using the Stratasys F900 machine (Eden Prairie, MN, USA). The diameter of the Ultem 9085 filament was 1.8 mm. A material tip T16 was applied, ensuring 0.254 mm layer height. The infill density was set to 100% (solid) for all samples, and the samples were printed at a raster width of 0.508 mm and a contour-to-raster air gap and raster-to-raster air gap of 0 mm. The dogbone specimens for tensile tests were printed with sizes according to standard ISO 527-1 (Type 1A) [25] in directions X, Y, and Z, as reported in [3]. The samples for the assessment of thermophysical properties with dimensions of approx. 5 × 5 × 5 mm were cut from a printed rectangular bar of 5 × 5 × 100 mm. The samples for optical smoke density and toxicity tests had dimensions of 75 × 75 mm according to Federal Aviation Regulation (FAR) and EASA Certification Specifications (CS) 25.853 [26]. The scheme of all test samples is provided in Figure 1, while photos of the samples tested in tensile and thermomechanical analysis tests in all printing layer orientations are provided in Figure 2. 

### 2.2. Tensile Tests

Tensile tests were performed using a Zwick 2.5 universal testing machine (Zwick Roell Group, Ulm, Germany) at a 5 mm/min strain rate and room temperature (RT) of 20 °C according to standard [25]. The fracture surfaces of the dogbone test samples were analyzed using a Canon EOS 40D digital SLR camera (Canon Inc., Shimomaruko, Tokyo, Japan). Isometric photographs of the fractured surfaces were captured for the samples printed in different printing orientations. Five test samples per printing layer orientation were tested for tensile tests, and the values corresponded to their arithmetic mean value.

### 2.3. Testing of Thermophysical Properties

The thermophysical properties of samples were investigated using the thermomechanical analysis expansion mode with Mettler-Toledo TMA/SDTA 841e (Greifensee, Switzerland). The measurements were carried out in three printing orientations: X, Y, and Z. Each sample was tested once for each orientation. The samples underwent two heating/cooling cycles at a temperature range from 30 to 190 °C with a heating rate of 1 °C/min. The glass transition temperature (*T*_g_) and linear thermal coefficient (CLTE) were defined on the second cycle, and the residual strain (ε) on the first cycle.

Differential Scanning Calorimetry (DSC) tests were performed for Ultem 9085 printed samples and filaments by heating the samples from RT to 250 °C, cooling to RT, and subsequent heating to 250 °C. For DSC tests, the glass transition temperature was evaluated from the second heating cycle to reduce the effect of thermal relaxation processes on the results obtained. Due to lower data scattering, three samples were used for the thermophysical tests.

### 2.4. Smoke Density and Toxicity Tests

Smoke density and toxicity tests were conducted at Lantal Textiles AG (Langenthal, Switzerland) using the National Bureau of Standards (NBS) Smoke Chamber following the requirements of FAR/CS 25.853 [26] and Airbus standard AITM3-0005 [27] for toxicity tests. Before testing, all specimens were conditioned at a temperature of 22.5 °C and relative humidity of 50% for at least 24 h to ensure consistency.

During the tests, the percentages of light transmission and optical density were recorded as a function of time (in minutes). The specific optical density was calculated to provide a dimensionless measure of the smoke produced per unit area of the material under flaming conditions. The maximum specific optical density observed within the first 4 min of testing was reported. In parallel, gas analysis was performed using detector tubes, with results compared to aviation industry toxicity requirements. A heat flux of 2.5 W/cm^2^ was applied during the tests.

The specific optical density must not exceed 200 to pass the smoke density test. For toxicity, the following thresholds were applied: hydrogen cyanide (HCN) ≤ 150 ppm, carbon monoxide (CO) ≤ 1000 ppm, nitrogen oxides (NO/NO₂) ≤ 100 ppm, sulfur dioxide (SO_2_) ≤ 100 ppm, hydrogen fluoride (HF) ≤ 100 ppm, and hydrogen chloride (HCl) ≤ 150 ppm. For each sample configuration, three specimens were tested. The reported values represent the arithmetic mean of the results obtained.

## 3. Results and Discussion

### 3.1. Morphology of Fracture Surfaces

**Figure 3:**
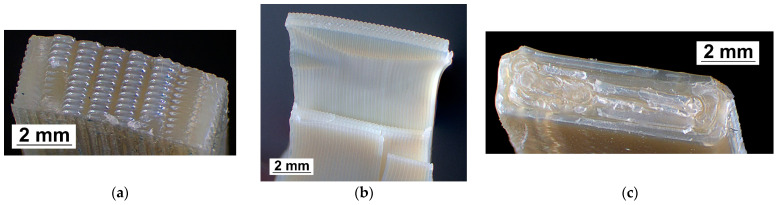
Fracture surfaces of PEI samples printed in (**a**) X, (**b**) Y, and (**c**) Z printing orientations.

The morphology of the fracture surfaces for transverse cross-sections of Ultem 9085 samples (X, Y, and Z) after tensile tests was analyzed by optical microscopy, and the results are provided in Figure 3. According to Figure 3a,b, the X and Y samples exhibited relatively smooth fracture surfaces because the loading direction was aligned with the filament orientation, and fracture occurred through filament breakage. Additionally, microscopic analysis revealed that the void distribution in the transverse cross-sections of samples printed in the X and Y directions was nearly identical, suggesting a similar level of fiber-to-fiber fusion. In contrast with the sample printed in the X orientation, the samples in the Y orientation displayed significant plastic deformation under tensile loading, leading to the formation of a “neck” and intralayer breakage (see Section 3.2. Tensile properties).

However, the samples printed in the Z orientation showed distinct transverse cross-sections with brittle filament structures. These characteristics suggest internal defects such as voids and inconsistent filament diameters, likely resulting from the anisotropic nature of FFF [8,24]. Therefore, mechanical failure occurred along the interlayer interfaces for these samples, as evidenced by the rough fracture surface showing filament peeling and pull-out [28]. Interlayer adhesion played a dominant role in the fracture process, leading to the lowest tensile strength, elastic modulus, maximal deformation, and highest thermal expansion observed among the samples printed in the Z direction (refer to Section 3.2., Tensile Properties, and Section 3.3., Thermophysical Properties).

Similar findings were observed regarding the fracture behaviour of Ultem 9085 samples with different printing layer orientations [5,29]. The X and Y samples exhibited the best tensile properties because the filaments were aligned parallel to the sample axes and in the same direction as the applied load. This alignment allowed these samples to resist the applied load effectively. In contrast, the fracture surface of samples printed in the Z orientation differed significantly, revealing debonding between layers and insufficient adhesion to withstand high loads [30]. Consequently, debonding at interfaces perpendicular to the tensile load was identified as the primary reason for the lower tensile properties, including reduced strength and elongation at break [5,29].

### 3.2. Tensile Properties

The material’s tensile strength was defined as the maximal stress attained by the specimen, while the tensile modulus was determined from the slope of a secant line drawn between 0.05% and 0.25% strain on the stress–strain curve.

**Figure 4:**
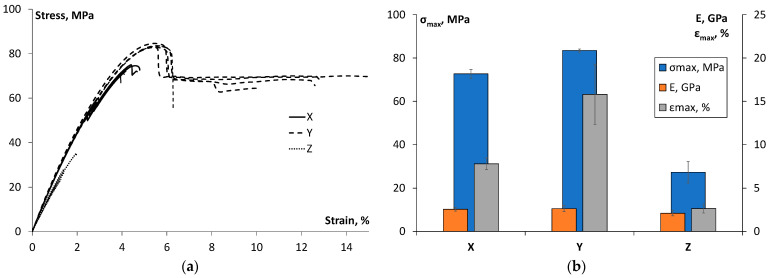
(**a**) Stress–strain curves and (**b**) tensile strength, elastic modulus, and maximal deformation for Ultem 9085 samples printed in different orientations (X, Y, Z).

All stress–strain curves for all printing orientations are shown in Figure 4a. The tensile test data provided in Figure 4 were not manually corrected for machine deflection, but Zwick software TestXpertIII (Zwick Roell Group, Ulm, Germany) provided a built-in compensation and automatic correction of travel measurements originating from the load cell and the test frame. Moreover, a clip-on extensometer was used for the initial tensile data to evaluate the tensile modulus at a higher precision. Since no notable changes in stress–strain curves were visible in Figure 4 after removing the extensometer, it could be assumed that the built-in compensation was satisfactory. The data scattering within one printing orientation was not substantial. The stress–strain curve analysis demonstrated a significant impact of printing direction on mechanical behaviour. According to Figure 4a, the samples printed in the Y direction exhibited notable plastic deformation and the development of a “neck”. Tensile strength, elastic modulus, and maximum deformation were evaluated for each printing orientation; the results are summarized in Figure 4b.

Figure 4b shows that the highest tensile strength and elastic modulus values were achieved for the samples printed in the Y direction. Conversely, the samples printed in the Z direction exhibited the lowest values, with tensile strength approximately 3 times lower and elastic modulus about 1.3 times lower. The samples printed in the X orientation achieved a 14.8% lower tensile strength than those printed in the Y orientation. Thus, the tensile strength for the X, Y and Z, printing directions was 72.7 ± 1.9 MPa, 83.5 ± 0.7 MPa, and 27.3 ± 4.9 MPa. The elastic modulus for the X, Y, and Z samples was equal to 2.6 ± 0.2 GPa, 2.6 ± 0.3 GPa, and 2.1 ± 0.3 GPa. According to Figure 4, the main effect of printing orientation was observed for maximal deformation: 7.8 ± 0.7% (X), 15.8 ± 3.5% (Y), and 2.6 ± 0.5% (Z).

The results obtained for all these characteristics reasonably correlated to the material datasheet provided by Stratasys [18] and previously reported results [5,8,24]. The difference in tensile characteristics observed between samples printed in the X and Y directions and those printed in the Z direction can be attributed to the longitudinal filament orientation in the X and Y directions, which aligns with the tensile load application direction [3,31,32]. The highest maximal deformation and tensile strength of samples printed in the Y orientation could be explained by the highest contour/infill area ratio amongst the samples, because the contour is on the longest side of the rectangular cross-section [5].

### 3.3. Thermophysical Properties

#### 3.3.1. Thermomechanical Analysis

**Figure 5:**
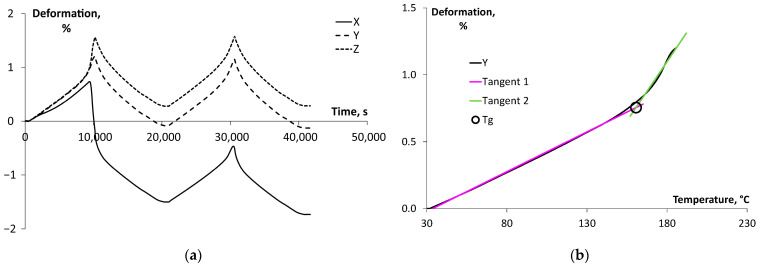
(**a**) Representative thermal curves for Ultem 9085 samples printed in different orientations (X, Y, Z) and (**b**) thermal deformation vs. temperature for Ultem 9085 tested in the Y printing orientation.

Thermomechanical analysis was performed on Ultem 9085 samples in three perpendicular axes, X, Y, and Z, where the *X*-axis was directed along the filaments, the *Y*-axis transverse to it, and the *Z*-axis normal to the X- and Y-axis plane. Thermal deformation (%) was defined as the ratio of change in the original height of the sample, and it is shown in Figure 5a as a function of time for Ultem 9085 samples tested in the X, Y, and Z orientations. The anisotropy of thermal behaviour is observed, revealing significant shrinkage (approx. −1.7%) for samples tested in the X orientation for both heating–cooling cycles. Obviously, printing orientation X (along the extruded filaments) caused notable thermal stresses in the printed parts, which were reduced due to heating and subsequent thermal relaxation. According to Figure 5a, the thermal curves for the rest of the printing orientations (Y and Z) look similar and without shrinkage, meaning that the transverse plane of the extruded filaments does not have significant anisotropy.

According to standard [33], the coefficient of thermal expansion (CTE) of plastics evaluated from TMA data is the tangent for thermal deformation as a function of temperature. In the case of a test specimen exhibiting a glass transition, CTE shall be calculated before and after the glass transition. Figure 5b shows the thermal deformation as a function of temperature for the samples tested in the Y printing orientation and for the second heating cycle, along with two tangents before and after the glass transition of Ultem 9085. Then, the glass transition temperature (*T*_g_) is determined as the intersection point of these tangents drawn to the TMA curve before and after the transition. The results obtained for the CTE of Ultem 9085 tested in the three orthogonal axes are summarized in Table 1.

According to Table 1, the CTE was the lowest before and after the glass transition temperature for the X printing orientation, revealing better thermal stability. In the perpendicular plane (Y and Z printing orientations), the results were 13–17% and 20–30% higher for the temperature intervals below and above the glass transition. Moreover, the glass transition temperature estimations show that in the X printing orientation, *T*_g_ is 171 °C; in the Y direction, 164 °C; in the Z direction, 165 °C. The residual deformation in the X direction (ε_x_) was −1.53%, in the Y direction, ε_y_ = −0.08%, and in the Z direction, ε_z_ = 0.28%. The residual strain values in each printing orientation (X, Y, Z) play an important role in understanding and optimizing the performance of 3D-printed materials. Residual strain refers to the strain remaining in a material after the applied load or thermal process (such as cooling after 3D printing) is removed. Residual strain significantly affects mechanical properties like toughness, fatigue life, fracture resistance, and also visco-elastic properties (e.g., creep) [34]. The samples tested in the X printing orientation demonstrated the highest thermal stability and glass transition temperature. The anisotropy observed in the thermal expansion and the relatively high thermal expansion of Ultem 9085 can be attributed to a higher disorder of molecular chains than in semicrystalline polymers [35]. Moreover, insufficient adhesion between the layers in printing orientation Z (vertical) during the deposition of molten polymeric layers was also reported as one of the main reasons for diminishing thermophysical performance [8,36,37].

#### 3.3.2. Differential Scanning Calorimetry

**Figure 6:**
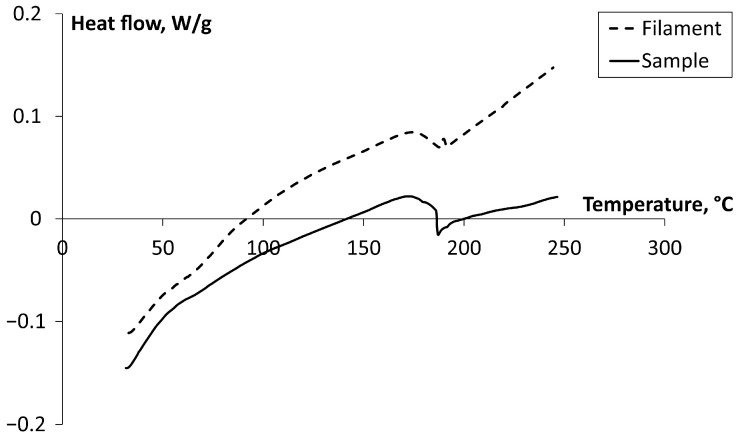
DSC curves for the Ultem 9085 printed sample and filament for the second heating cycle as indicated on the graph.

Using this method, the difference in the heat needed to raise the temperature of a sample as compared to the reference is evaluated as a function of temperature. Therefore, due to the specificity of this method, it is impossible to evaluate the effect of printing layer orientation on the thermal curves or glass transition temperature estimated by using it. This method was used to compare the results of TMA in general. The DSC curves of the second heating cycle for the Ultem 9085 printed sample and filament (PEI and PC) are shown in Figure 6. The DSC curves reveal common glass transition processes at the 170–200 °C temperature interval.

The glass transition temperature of the Ultem 9085 sample and filament was evaluated following the standard ISO 11357-1:2023 procedure for determining midpoint temperature [38]. The glass transition midpoint temperature for the printed sample and filament of Ultem 9085 is, consequently, 183 °C and 184 °C. This shows that no significant changes occurred in the PEI macromolecular structure. The results for *T_g_* using DSC are 12–18 °C higher compared to TMA, but since it is another thermal method, such variation can be expected to occur. According to the material datasheet by Stratasys [13], the glass transition temperature evaluated by DSC for the second heating–cooling cycle is 177 °C, which is very close to the results obtained. The glass transition temperature for PEI, PC, and their blends (5–40 wt.% of PC) was investigated using DSC in [39] and the *T*_g_ reported for PEI and PC was 208 °C and 147 °C, respectively.

### 3.4. Smoke Density and Toxicity Characteristics

**Figure 7:**
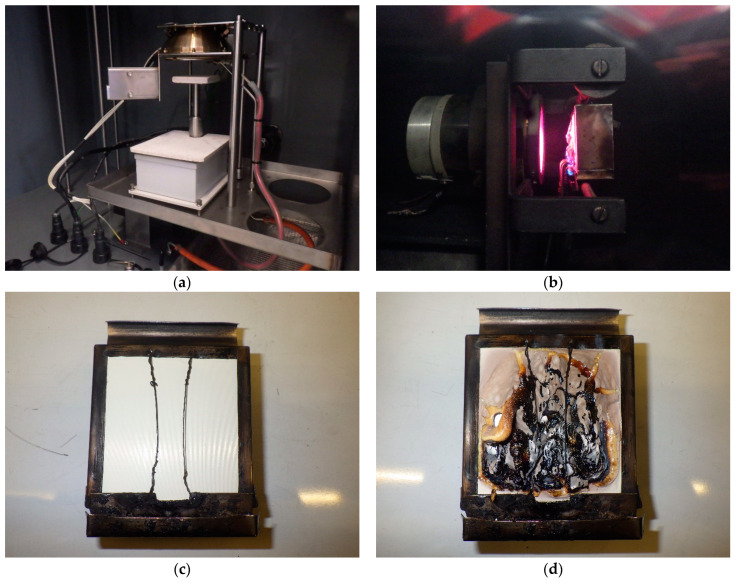
Photos of (**a**) NBS smoke chamber, (**b**) burner, and Ultem 9085 samples (**c**) before and (**d**) after smoke density and toxicity testing.

Figure 7 provides photos of the testing chamber (a) and burner (b) used for smoke density and toxicity tests, along with the 75 × 75 mm Ultem 9085 samples before (c) and after (d) the testing.

**Figure 8:**
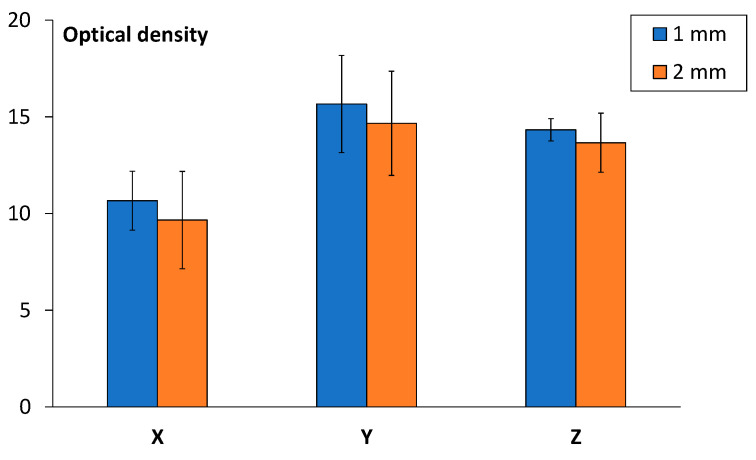
Optical density after 4 min for samples printed in the X, Y, and Z orientations at 1 mm and 2 mm thicknesses.

The smoke density test results for samples printed in the X, Y, and Z orientations at different thicknesses (1 mm and 2 mm) are presented in Figure 8. As previously noted, the optical density must not exceed 200 to meet regulatory requirements. According to the data in Figure 8, all 3D-printed samples satisfied this criterion, confirming their compliance with standards outlined in FAR 25.853, making them suitable for aircraft interior applications. Considering the sample geometry, the Y and Z print orientations are regarded as equivalent due to the symmetry of the specimens. While the X printing orientation results were slightly lower, the overall effect of print orientation was minimal and can be considered insignificant. Figure 8 also illustrates that the smoke density values were consistent across all build directions. This finding aligns with observations from the 60 s vertical burn test, where flammability properties were similarly independent of print orientation, as reported previously [4].

**Figure 9:**
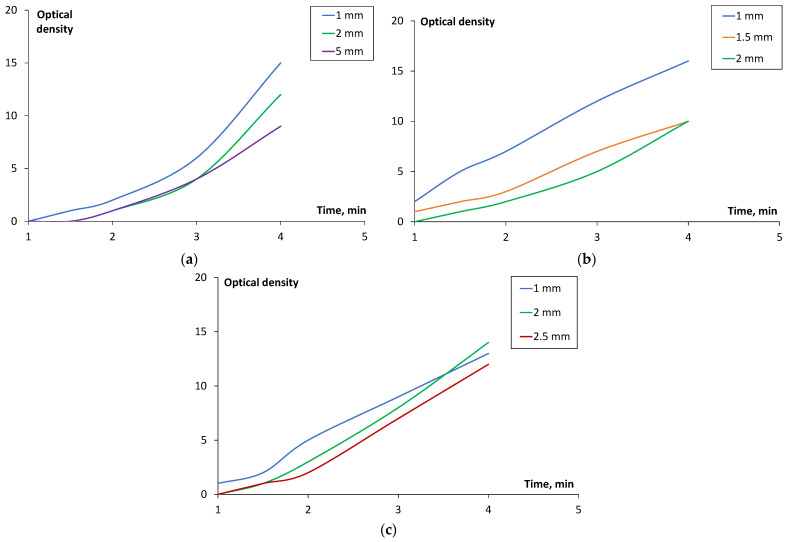
Optical density vs. time for samples printed in the (**a**) X, (**b**) Y, and (**c**) Z orientations.

Although requirements for smoke density are defined 4 min after ignition, the dynamic test results for smoke density in time, which consider both print orientation and sample thickness, are shown in Figure 9, where (a) shows smoke density results for samples printed in the X, (b) Y, and (c) Z orientations at different thicknesses. The smoke density of a 3D-printed part can vary based on printing orientation due to differences in microstructure, porosity, and interlayer adhesion. Horizontal prints (X-oriented specimens in this paper, Figure 1) could produce less smoke due to better structural integrity and lower porosity. The layers are oriented in-plane for this printing orientation, leading to stronger layer adhesion and potentially lower thermal decomposition rates. Vertical prints (Y- and Z-oriented specimens in this paper, Figure 1) typically have weaker interlayer bonds compared to the horizontal direction, resulting in easier thermal degradation under fire and higher smoke generation. The vertical orientation may also hypothetically introduce voids or anisotropic structures that trap and release gasses differently during combustion. Samples printed in the Y and Z direction at 1 mm thickness exhibited a slightly higher smoke density than the 2 mm samples. The general results follow the trend that the higher the thickness of the sample, the lower the optical smoke density.

The results from the toxicity tests are summarized in Table 2. Generally, all samples tested did not exceed the limits and efficiently met the requirements, highlighting the suitability of Ultem 9085 as a robust and high-performing material for critical applications in the aviation industry. These results align closely with Stratasys’ material specifications and performance data [18]. While Stratasys specifications do not specify the tested thickness, the data from this study indicate that print orientation and specimen thickness have minimal influence on toxicity performance. No comparable toxicity data for Ultem 9085 were identified in the available literature.

## 4. Conclusions

This study aimed to investigate the anisotropic effects introduced by the FFF process on the tensile, thermophysical, smoke, and toxicity properties of Ultem 9085 (PEI and PC) by testing samples printed in three orthogonal printing orientations (X, Y, and Z).

The results obtained for the tensile tests indicate that the Y printing orientation exhibited the highest tensile strength and maximum deformation. While the properties of the Y-oriented samples were not significantly different from those of the X-oriented samples—except for the elastic modulus, which was comparable in both orientations—the Z-oriented samples showed the lowest tensile properties. This reduction is primarily attributed to fractures caused by interlayer debonding, as the adhesion between the layers is insufficient to withstand high tensile loads. These findings emphasize the strong mechanical performance of Ultem 9085 based on the printing orientation, particularly in the X and Y directions, highlighting the significant impact of building direction on its tensile properties.

The results for the thermophysical properties indicate that the samples tested in the X printing orientation exhibited the highest thermal stability, characterized by the lowest coefficient of thermal expansion (CTE) and the highest glass transition temperature. The observed anisotropy in thermal expansion and the relatively high thermal expansion of Ultem 9085, a blend of two amorphous materials (PEI and PC), can be attributed to a more significant disorder among the molecular chains than in semicrystalline polymers. The glass transition temperature for the Ultem 9085 printed sample and filament evaluated by DSC is 12–18 °C higher compared to TMA, but since it is another thermal method, such variation can be expected to occur.

The analysis of Ultem 9085 samples in terms of smoke density and toxicity demonstrates the material’s substantial compliance with aviation safety standards. The smoke density test confirmed that all tested samples, regardless of print orientation or thickness, remained below the regulatory limit of 200, affirming their suitability for aircraft interior applications as per FAR 25.853. Additionally, toxicity tests confirmed the material’s compliance with safety thresholds. While Stratasys specifications provide baseline performance metrics, this study fills a gap by illustrating the negligible impact of print parameters on toxicity and smoke density performance. Notably, no additional smoke and toxicity data for Ultem 9085 were found in the literature, underscoring the significance of this research.

## Figures and Tables

**Table 1:** The values of CTE in three printing orientations at temperatures below and above *T*_g_.

Interval	X	Y	Z
Below *T*_g_	5.53 × 10^−5^	6.26 × 10^−5^	6.49 × 10^−5^
Above *T*_g_	1.38 × 10^−4^	1.65 × 10^−4^	1.8 × 10^−4^

**Table 2:** The results of toxicity testing.

Orientation	Thickness, mm	HCN, ppm	CO, ppm	NO/NO_2_, ppm	SO_2_, ppm	HF, ppm	HCl, ppm
X	1.50	<1	124.0	<1	1	<1	<1
X	2.00	<1	105	<1	<1	<1	<1
X	2.50	<1	110.7	<1	<1	1	<1
X	5.00	<1	93.7	<1	1	<1	<1
Y	1.00	<1	127.0	<1	1	<1	<1
Y	1.50	<1	103.3	<1	<1	<1	<1
Y	2.00	<1	113.7	<1	<1	<1	<1
Z	1.00	<1	111.7	<1	<1	<1	<1
Z	2.00	<1	107.3	<1	1	<1	<1
Z	2.50	<1	111.7	<1	1	<1	<1

## Data Availability

The data presented in this study are available on request from the corresponding author.
